# Applying Discrete Event Simulation to Reduce Patient Wait Times and Crowding: The Case of a Specialist Outpatient Clinic with Dual Practice System

**DOI:** 10.3390/healthcare10020189

**Published:** 2022-01-19

**Authors:** Weng Hong Fun, Ee Hong Tan, Ruzelan Khalid, Sondi Sararaks, Kar Foong Tang, Iqbal Ab Rahim, Shakirah Md. Sharif, Suhana Jawahir, Raoul Muhammad Yusof Sibert, Mohd Kamal Mohd Nawawi

**Affiliations:** 1 Institute for Health Systems Research, National Institutes of Health, Ministry of Health Malaysia, Shah Alam 40170, Malaysia; jdreehong@moh.gov.my (E.H.T.); sararaks.s@moh.gov.my (S.S.); tangkarfoong@gmail.com (K.F.T.); fathullah@moh.gov.my (I.A.R.); shakirah.ms@moh.gov.my (S.M.S.); suhana.j@moh.gov.my (S.J.); 2 Institute of Strategic Industrial Decision Modelling, School of Quantitative Sciences, Universiti Utara Malaysia, Sintok 06010, Malaysia; ruzelan@uum.edu.my (R.K.); mdkamal@uum.edu.my (M.K.M.N.); 3 Hospital Pakar Sultanah Fatimah, Muar 84000, Malaysia; raoul@moh.gov.my

**Keywords:** wait times, discrete event simulation, dual practice, outpatient, arrival pattern, crowding

## Abstract

Long wait times and crowding are major issues affecting outpatient service delivery, but it is unclear how these affect patients in dual practice settings. This study aims to evaluate the effects of changing consultation start time and patient arrival on wait times and crowding in an outpatient clinic with a dual practice system. A discrete event simulation (DES) model was developed based on real-world data from an Obstetrics and Gynaecology (O&G) clinic in a public hospital. Data on patient flow, resource availability, and time taken for registration and clinic processes for public and private patients were sourced from stakeholder discussion and time-motion study (TMS), while arrival times were sourced from the hospital’s information system database. Probability distributions were used to fit these input data in the model. Scenario analyses involved configurations on consultation start time/staggered patient arrival. The median registration and clinic turnaround times (TT) were significantly different between public and private patients (*p* < 0.01). Public patients have longer wait times than private patients in this study’s dual practice setting. Scenario analyses showed that early consultation start time that matches patient arrival time and staggered arrival could reduce the overall TT for public and private patients by 40% and 21%, respectively. Similarly, the number of patients waiting at the clinic per hour could be reduced by 10–21% during clinic peak hours. Matching consultation start time with staggered patient arrival can potentially reduce wait times and crowding, especially for public patients, without incurring additional resource needs and help narrow the wait time gap between public and private patients. Healthcare managers and policymakers can consider simulation approaches for the monitoring and improvement of healthcare operational efficiency to meet rising healthcare demand and costs.

## 1. Introduction

Malaysia has a hybrid healthcare system consisting of public and private healthcare delivery systems. The public healthcare system has provided near-universal access to quality healthcare services for free or with a nominal fee over the years despite low levels of government financing for health [1]. In contrast, the private healthcare system is funded primarily by out-of-pocket payments by patients who choose to seek private healthcare services. The growth of the private healthcare sector has partly contributed to the issue of brain drain of skilled health workforce from the public healthcare sector, creating a need for policy changes to address this, with one of them being the introduction of dual practice within the public healthcare system.

Dual practice in public hospitals has been established in Malaysia since 2007 with an overarching objective to retain specialists serving under the Ministry of Health (MOH) Malaysia. Senior specialists who wish to provide private services on top of public services would receive remuneration through fee-for-service in addition to their fixed monthly salaries. Additional revenue generated from private services would also be channelled to the government [2]. Under the dual practice regulations, specialists are restricted to providing private services after the completion of public service provision to ensure that private services do not affect public care provision and resource use [2]. Nevertheless, the existence of different patient queues as well as sharing of public healthcare facilities and resources among public and private patients in dual practice settings may inadvertently affect healthcare service access and efficiency if services are not well-regulated [3,4,5].

The impact of dual practice on outpatient care access among public and private patients in MOH hospitals offering the service is, however, still largely unknown. Long wait times for public patients are thought to be accentuated in dual practice systems [3,4,6], which undermines equitable access to care due to faster access for patients with the ability to pay for private services [7]. This indicates the need to understand the impact of dual practice on wait times for both public and private patients in the case of Malaysia. Another challenge is that within Malaysia’s public healthcare system at large, long wait times and crowding continue to be barriers to timely and efficient outpatient care provision, despite commendable improvements in healthcare access [8]. These barriers have been attributed to the increase in healthcare utilisation as well as the scarcity of health funding and resource allocation [8,9]. Long wait times can lead to unnecessary delays in care, poorer perceived quality of care, and lower patient satisfaction [10,11,12,13], which highlights the importance of wait time improvement to ensure optimal service delivery.

Resource-constrained healthcare policymakers face hard choices in determining effective and efficient strategies for wait time improvement without inadvertently increasing cost or burden to the public healthcare system. This is due to the inherent complexity in healthcare systems [14], and recommendations to improve outpatient services would need to be tested and deliberated on before implementation to prevent potential unintended effects on operational processes, performance measures, and service provision. As such, there is a potential for the use of operational research techniques to aid in healthcare decision-making [15,16].

Discrete event simulation (DES) is an operational research technique that can be used to simulate behaviours of complex systems and create models that represent real-world systems with the incorporation of resource constraints and entities with specific attributes [17,18]. Operational characteristics in the model can be changed by conducting scenario analyses to evaluate possible impacts from strategies and identify the most effective strategy without changing the actual system. DES has been used to study patient or operational flow, patient appointment scheduling, resource allocation, and capacity planning to evaluate outcomes on wait times, service performance, and resource utilisation [15,17,19,20,21,22,23,24,25,26,27,28].

Although there have been numerous DES studies on patient arrival/healthcare provider scheduling strategies to reduce wait times, such as by changing patient appointment or service/provider schedules [29,30,31,32,33,34,35] or implementing even distribution of arrival pattern/consultation slots [36,37], these studies have mainly focused on single patient groups. It is still unclear how such wait time improvement strategies will impact public and private patients in dual practice settings. Thus, this study aims to evaluate the effects of changing consultation start time and patient arrival on wait times and crowding in an outpatient clinic with a dual practice system using a DES approach.

## 2. Materials and Methods

A DES model was developed to understand the impact of changing consultation start time and patient arrival on outpatient wait times and crowding. The simulation process involved real-world data collection for model data input, base case DES model building and validation, as well as scenario testing. DES was selected instead of other modelling techniques such as system dynamics because the problem under study involves constrained resources [18]. The model would allow for random variation in inputs over time, as entities change in state and flow through queues and activities [38,39,40]. Entities can also be modelled at the individual level with variability in arrival and service times [33].

### 2.1. Setting

This study was conducted in a Malaysian public tertiary hospital in 2017. The Obstetrics and Gynaecology (O&G) specialist outpatient clinic within the hospital was selected as the study site as it served the overall highest annual number of private patients in the hospital as of the year when the study was conducted. The clinic provides prenatal, early pregnancy assessment, antenatal, postnatal, general gynaecology, contraception, and infertility care services, where patients can choose to seek care as a public or private patient. Private patients pay (fee-for-service) to access specialist care provided by their preferred specialists under the dual practice system in the study hospital, while public patients access healthcare services with nominal to no charge but do not have the option to choose their doctors.

### 2.2. Operational Characteristics and Patient Flow

Discussions were held with hospital managers, O&G clinic administrative staff, and healthcare providers to understand resource availability, patient load, patient flow, and clinic processes. The clinic has ten consultation rooms, two nurses’ stations, one registration counter, two vital sign measurement stations, a laboratory, and a general waiting area. Human resources include doctors, specialists who provide only public services or both public and private services, nurses, healthcare assistants, and administrative staff. Approximately seven doctors and three specialists are available to see patients with appointments (new and follow-up cases) or without an appointment (walk-ins with referral) from 8:00 a.m. to 1:00 p.m. on working days, while the number of doctors available after 1:00 p.m. is dependent on clinic schedule and patient load. Private patient registration typically starts after 11:30 a.m., and private services are provided by two specialists from Monday to Thursday. An average of 86 public and seven private patients were seen on a typical working day (October 2017) (Table S1).

**Figure 1:**
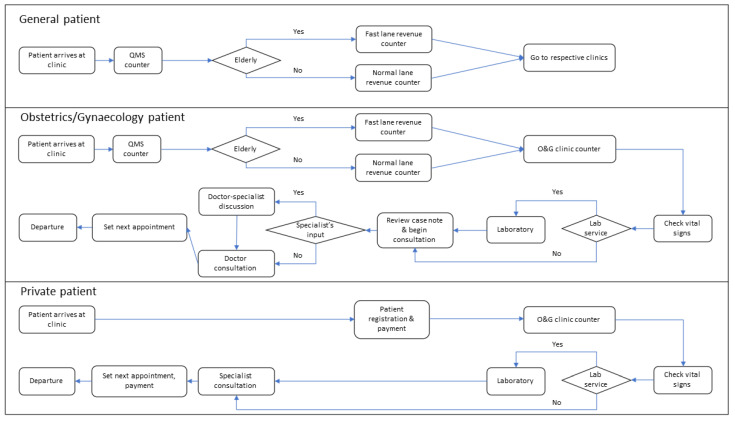
Flow diagram for patients at the outpatient department and O&G clinic. Note: General patients: Patients who attend outpatient clinics other than O&G clinic (non-O&G services) and share the same QMS and revenue counters with O&G patients. Obstetrics patients: Patients obtaining obstetrics services at the O&G clinic. Gynaecology patients: Patients obtaining gynaecology or fertility services at the O&G clinic. Private patients: Patients obtaining private obstetrics or gynaecology services at the O&G clinic. Lab: laboratory; QMS: general outpatient registration counter.

The public patient flow starts at the outpatient department, with patients getting a queue number at the general (QMS) counter for outpatient registration at the study hospital starting at 7:00 a.m. Patients are required to register and pay for services at the revenue counters before proceeding to their respective clinics. The QMS and revenue counters are shared among specialist clinics, including O&G (public Obstetrics (Obs) or Gynaecology (Gyn) patients), paediatrics, otorhinolaryngology, ophthalmology, and general medicine (general patients). Patients then proceed to the O&G clinic counter to register and obtain an O&G clinic queue number. Patients must undergo vital sign screening at the vital sign measurement station before consultation and proceed to the laboratory if required. Patients then wait for consultation. Once consultation is completed, patients obtain new appointment dates at the nurses’ station before leaving the clinic. The private patient flow differs from public patients in that they register at a designated private patient counter after 11:30 a.m. before proceeding to the O&G clinic. This designated counter serves as a registration, appointment, and revenue counter for private patients. Clinic processes are similar to that of public patients except for specialist-only consultation. They need to return to the designated counter for payment and appointment after consultation. An overview of patient flow based on patient type is shown in Figure 1.

#### 2.2.1. Time-Motion Study (TMS)

We conducted a TMS at the outpatient department and O&G clinic in October 2017, collecting data on time taken for registration and clinic processes at each location (station). Given the resource-intensive procedure, the TMS was divided into two parts:

1. Observation 1 involved the recording of the time taken for public patient registration processes at the QMS counter, revenue counters, and O&G clinic counter over five working days. Due to fast processing times and multiple patients checking in concurrently at QMS and revenue counters, we randomly sampled patients at different time intervals during the TMS.
2. Observation 2 involved the recording of the time taken for clinic processes for public and private patients at the O&G clinic counter, vital sign measurement station, laboratory, consultation rooms, and nurses’ station as well as registration and appointment processes at the private patient counter over five working days. Patients’ medical record number was recorded at the O&G clinic counter.

#### 2.2.2. Time Required for Case Review and Management

Discussions with hospital managers, doctors, and specialists involved obtaining information on work schedules, estimates of time for electronic medical record (EMR) review, documentation, consultation for follow-up/new cases, and case discussion between doctors and specialists as these processes are highly variable, case-specific, and could not be tracked during TMS. Approximately 10% of daily cases seen by doctors required a specialist discussion/consultation.

### 2.3. Data Management

Data required for this study were extracted and provided by medical record officers from the study hospital. Data were extracted from the study hospital’s Total Hospital Information System (THIS) database, and these include patient medical record number, age, gender, discipline, type of service and patient (follow-up or new patient), appointment booking date and time, arrival times, and electronic registration time at the revenue counter. Patients’ medical record number in THIS and TMS data were linked to (1) exclude non-O&G clinic patients at the QMS and revenue counters, and (2) consolidate arrival times, process times (difference between the start and end times at each location), and time in between each process for each O&G patient observed during the TMS. Patients with missing data, data inconsistency, standard process flow deviation (e.g., obtaining laboratory services after consultation, multiple laboratory visits), atypical consultation times (multiple/long consultation sessions), registration time anomalies, or who only had laboratory services (i.e., did not have a doctor consult session) were excluded from analysis (Figures S1 and S2). Both THIS and TMS data were used to determine the turnaround time (TT) from arrival to O&G counter registration (registration TT) and from O&G counter registration to departure (clinic TT). TT includes both process times and patient wait times at the O&G clinic.

### 2.4. Simulation

The applied simulation method was DES. The DES model was built using FlexSim Healthcare software (FlexSim Software Products, Inc., Orem, UT, USA, version 5.3.10). We created a replica model of QMS, private patient, and revenue counters at the outpatient department and the O&G clinic using the software. Operational characteristics and patient flow as described in Section 2.2 were simulated in our DES model. Reporting of DES study was conducted according to the Strengthening the Reporting of Empirical Simulation Studies (STRESS-DES) guidelines [41]. Its use has been reported in other healthcare DES studies [33,40].

Arrival times of general and O&G patients from THIS, process times from TMS, as well as time taken for EMR review and consultation from expert opinion were used as input data to fit the best probability distributions for each patient type and to reflect real-world process times in the model using FlexSim’s ExpertFit software. Detailed input parameters are available in Table S2. As the focus of the simulation was on wait time and crowding at the O&G clinic, patient arrival data during TMS Observation 2 period were used for simulation. A total of 4802 patients with complete arrival time and patient type data were included in the simulation (Table S3). They were divided into general (non-O&G), public Obs, public Gyn, and private patients, and were modelled as entities with attributes based on age (elderly, non-elderly) and appointment type (follow-up/new case). General patients were modelled only at the QMS and revenue counters. Other entities include healthcare providers and administrative staff. O&G clinic scheduling rules such as private patient ratio per specialist were also included. Patients arrive according to real-world block arrival patterns, wait/queue when resource constraints occur during registration or clinic processes, and exit from the system at the end of clinic processes at the nurses’ station or private patient counter. Block arrival pattern was characterised by a random number of patient arrivals at any time point, with a large number of arrivals during peak hours. A random distribution of patient arrival was used in the base case model, and patients were simulated from arrival at the QMS counter to departure from the O&G clinic to determine the overall TT.

Assumptions set in the DES base case model include: (1) all patients visit each station once during their O&G clinic visit, (2) patients were served based on a first-in, first-out rule, (3) assignment to doctors is done randomly, (4) doctors work continuously until all patients are served, (5) nurses assisting doctors/specialists for procedures were not a constraint factor, and (6) one-hour lunch break was considered for specialists before serving private patients in the afternoon. The base case accounts for the time taken due to atypical consultation time and inactive consultation rooms (more than 15 min) during active clinic hours. Patients with atypical consultation times occupy consultation rooms for an extended period, rendering doctors and consultation rooms (resources) to appear constrained in the system during this period. A total of 30 independent replications were run to allow the sampling distribution to have an approximate normal distribution based on the Central Limit Theorem [42]. The length of each replication was five working days at the clinic. DES model layouts are available in Figures S3 and S4.

#### 2.4.1. Model Validation

Model face validation was conducted through stakeholder engagement discussions on DES model development with hospital managers to ensure that the model was a close representation of the O&G clinic system. Model validation also involved comparison of base case model outputs on clinic and registration TT against TMS findings before using the model for scenario analyses. Simulation of the base case model with random or even distribution of patient arrival produced similar output on overall TT, with a minimal difference of around three min (Table S4), thus allowing it to be used as the base case for scenarios with either random or even distribution of patient arrival.

#### 2.4.2. Simulation Experiment with Scenarios

Several scenarios (Table 1) were developed based on consensus among research team members to understand the effects of changing consultation start time and/or patient arrival on overall TT and patient crowd reduction. For example, based on the results from TMS, we matched consultation start time with staggered patient arrival time to evaluate the effect on overall TT. Changes in overall TT reflect the change in patient wait times at the O&G clinic as process times remained constant across all scenarios. Staggered patient arrival in scenario simulation is defined as an evenly distributed number of patients and arrival pattern for every 30-min slot. Each scenario simulation consisted of 30 replications. O&G patients were simulated from arrival at the QMS counter to departure from the O&G clinic. In comparison with the base case, an assumption made for all scenarios was that there is no atypical or prolonged consultation and inactive consultation room during active clinic hours to allow resources to be available for the consultation queue.

The number of patients for each 30-min slot was either seven or ten, based on resource availability, where there is a maximum of ten consultation rooms. The optimal number of consultation rooms was reduced to seven in one scenario (Scenario 5) to account for inactive consultation rooms as doctors might have other duties during clinic hours. There were no changes in resources and the total number of patients in the base case or scenario simulation. DES model results were generated from the software and exported as Excel files for analysis. Model outputs on overall TT and the number of patients waiting at the clinic area (crowd) per hour for each scenario were compared against that of the base case.

### 2.5. Statistical Analysis

We conducted Kruskal–Wallis H test to compare TMS process times and TT between patient types, as well as Mann–Whitney Test to compare consultation times between Obs public and private patients (as most of them were Obs patients). Data analyses were performed using IBM SPSS Statistics (version 26.0, IBM, Armonk, NY, USA).

## 3. Results

### 3.1. TMS Process Times and Turnaround Times

The median registration and clinic TT for private patients were significantly shorter (*p* < 0.01) compared to public Obs and Gyn patients due to the differences in registration processes and arrival times (Table 2), while consultation time was found to be significantly shorter for public compared to private patients. Details on patient selection for TMS observations were included in Figures S1 and S2.

### 3.2. Model Validation

**Figure 2:**
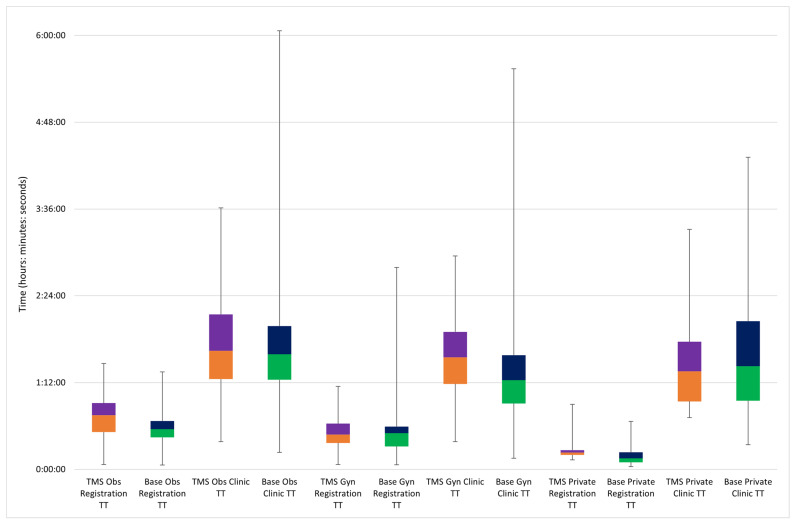
Box plot for comparison between base case outputs and TMS findings on registration and clinic TT for each patient type.

Figure 2 shows the comparison between base case outputs and TMS findings on registration and clinic TT for each patient type. The output values were close to those measured in the actual system. Face validation with hospital managers and research team members confirmed the close representation of the base case to the actual clinic system.

### 3.3. Effects of Scenarios on Overall TT and Number of Patients Waiting at the Clinic

**Figure 3:**
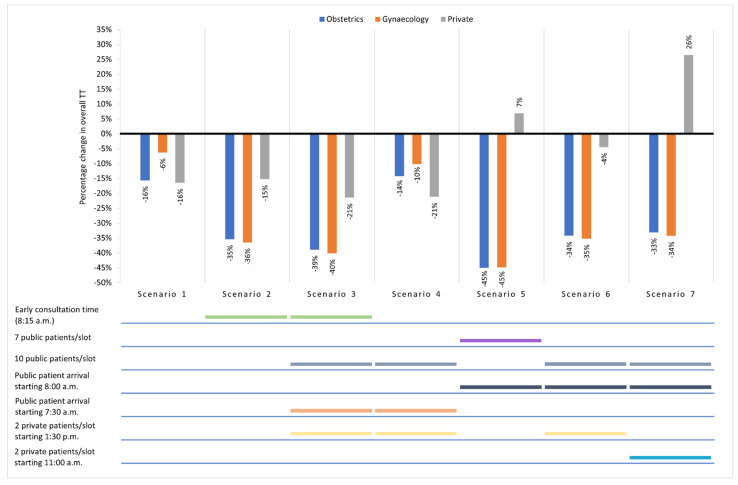
Percentage change in overall TT for Obs, Gyn, and private patients for each scenario in comparison with the base case (0%). Simulation outputs are available in Table S5.

Figure 3 shows the percentage change in (median) overall TT from the base case for public and private patients in each scenario. Reduction in overall TT was observed in Scenario 1 (even arrival pattern and typical consultation times). Earlier consultation start time for public patients resulted in overall TT reduction of up to 36% for public and 15% for private patients (Scenario 2). When consultation start time was adjusted to match registration start time and staggered arrival (ten public and two private patients per time slot), a greater reduction in overall TT was observed for both patient groups (maximum of 40% for public and 21% for private patients) (Scenario 3). However, early public patient arrival and staggered arrival without a congruent consultation start time (Scenario 4) resulted in the lowest overall TT reduction for public patients, although a reduction in overall TT for private patients (similar to that of Scenario 3) was still seen with staggered private patient arrival. Overall TT reduction of 45% for public patients can be achieved if the number of staggered arrivals is reduced to seven public patients per time slot and consultation sessions are extended till afternoon (Scenario 5), but there would be a slight increment in overall TT for private patients. Scenarios 6 and 7 led to similar overall TT reductions (up to 35%) for public patients when staggered arrival was simulated with public patients’ last slot set at 12:30 p.m. However, there was a minimal reduction in overall TT for private patients in Scenario 6, while early staggered arrival of private patients in Scenario 7 further increased the overall TT for private patients.

**Figure 4:**
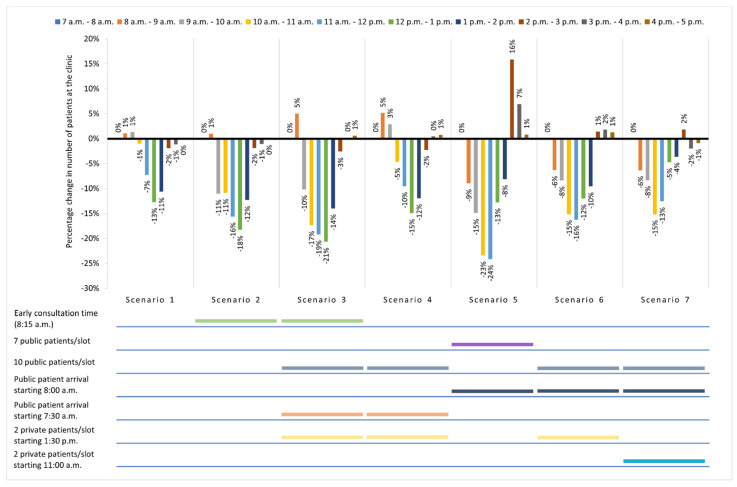
Percentage change in the number of patients at the O&G clinic waiting area per hour for each scenario in comparison with the base case (0%). Simulation outputs are available in Table S6.

Based on the percentage change in the (average) number of patients at the clinic waiting area at every hour from the base case (Figure 4), a similar trend was observed with overall TT reduction, where an incremental reduction in patient crowd was observed from 9:00 a.m. to 1:00 p.m. in Scenarios 1, 2, and 3. Scenario 4 led to a lower percentage of crowd reduction during peak hours (9:00 a.m. to 12:00 p.m.) compared to Scenarios 2 and 3 but had a similar increase in crowd percentage (5%) as with Scenario 3 from 8:00 a.m. to 9:00 a.m. due to early public patient arrival (7:30 a.m.). Although Scenario 5 resulted in the highest percentage of crowd reduction for public patients, the crowd percentage was 7 to 16% higher from 2:00 p.m. to 4:00 p.m. due to staggered public patient arrival and the shift in public patients’ last time slot (3:00 p.m.). Scenarios 6 and 7 led to similar changes in crowd percentage, where staggered public patient arrival resulted in better percentage of crowd reduction than without (Scenario 1) from 8:00 a.m. to 11:00 a.m., but with lower crowd percentage reduction from 12:00 p.m. to 1:00 p.m. due to private patient arrival in combination with ongoing public patient arrival (until 12:30 p.m.) or early private patient arrival (11:00 a.m.).

## 4. Discussion

The study showed that there were significant differences in TT between public and private patients, with private patients having considerably shorter overall TT. Depending on simulation configurations, each scenario would result in different levels of reduction in wait times and patient crowd. Timely consultation start time that is congruent with staggered arrival was found to result in the most optimal reduction in wait times and crowding for public and private patients at the O&G clinic without the need for additional resources.

The comparatively short process time at each station but high median registration and clinic TT during TMS for public patients might indicate that time was spent mostly on queuing and waiting for registration, as well as waiting after registration and in between clinic processes until the start of consultation. This was also reported in other studies [32,43], where wait times can be substantially greater than process times in settings with variability in patient arrivals, especially if utilisation of resources is close to maximum. Although it is recognised that private patients have different registration processes and arrival times, bulk arrival of public patients under block appointment scheduling enabled doctors to complete most public service provision before 1:00 p.m. and specialists to start private service provision in the afternoon. This might have led to significantly longer registration and clinic TT for public patients and crowding during peak hours but not for private patients due to their arrival after peak hours and the lower number of private patients compared to public patients. These reflect the need for effective strategies to bridge the wait time gap between public and private patients.

Reduction in overall TT by solely changing the consultation start time reaffirms that a part of the wait time and crowding issue is attributed to waiting for consultation after registration and other clinic processes among public patients (Scenario 2). Similarly, in other scenarios (Scenarios 3, 5, 6, and 7) where consultation start times were in concordance with staggered arrival time, reductions in overall TT are evident for public patients. In line with literature, delays in clinic start time negatively impact wait times as all appointments would be affected by late start time or doctors’/healthcare providers’ (un)punctuality [44,45]. However, a high patient load with block arrival will still require patients to wait, especially during peak hours regardless of consultation start time or arrival time as the limiting factors are the number of consultation rooms and doctors. While it may not be feasible to increase these resources in a resource-constrained setting, changing the number of patient arrivals to spread out patient load through staggered arrival can be an alternative solution. Scenario analyses showed that timely consultation start time which matches patient arrival, and implementation of staggered arrival (Scenario 3) would reduce overall TT for all patient types and contribute to patient crowd reduction during clinic peak hours. The greater reduction in wait times for public patients compared to that of private patients would help narrow the wait time gap between public and private patients. Early staggered arrival without a congruent consultation start time (Scenario 4) did not result in better reductions in overall TT and crowding for public patients, indicating that a combination of strategies is needed concurrently to obtain desired outcomes in a dual practice setting.

We noted that whilst configurations on staggered arrival would reduce overall TT for public patients, it would increase private patients’ overall TT (Scenarios 5 and 7). Private services are fairly optimised for private patients (e.g., shorter wait times, services start in the afternoon in outpatient settings) under the dual practice system at the time of the study [2], rendering staggered arrival for private patients less beneficial for them if public service provision is not somewhat completed before the start of private services. This may also explain why a timely consultation start time that matches staggered arrival time (for public patients) improves wait times for both patient types, creating a win-win situation. The increase in patient crowd from 2:00 p.m. onwards corroborates the increase in overall TT for private patients, further demonstrating the unfavourable impact on private patients’ overall TT if there is a delay in private service provision inflicted by slower clearance of public patients due to staggered arrival.

Crowd reduction at the O&G clinic, particularly during clinic peak hours was in parallel with overall TT reduction in all scenarios, suggesting that matching consultation start time and staggered patient arrival could also contribute to crowd reduction in addition to wait time reduction. Although this study did not simulate the effects of crowding at QMS and revenue counters that were shared among multiple specialist clinics, disproportionate patient arrival patterns for all patient types were observed (Table S3), with many of them queuing for registration early in the morning, and peak arrivals occurring between 8:00 a.m. to 10:00 a.m. The heavy patient flow (up to a maximum of 152 patients per 30 min) and crowding within a confined area might potentially lead to staff stress [46] and poor patient experience or satisfaction [47,48]. Additional research on strategies to mitigate such situations in outpatient settings would be useful for crowd reduction, especially when physical distancing is required amid the COVID-19 pandemic.

This study has several limitations. Patient no-shows or lack of punctuality, crowding caused by patients’ family member(s), time spent waiting before the start of QMS counter registration, and healthcare providers’ idle time were not included in the simulation. The inclusion of patient and healthcare provider behaviours in such simulation studies would provide a more comprehensive understanding of the effects of simulation configurations on wait times and crowding. Simulation of patient flow ends with patients’ departure from the O&G clinic/private patient counter, thus processes occurring beyond this point, such as pharmacy processes, were not simulated. The exclusion of patients with process flow deviations or multiple consultation/laboratory services may result in a more efficient system in the base case model compared to the real-world situation, which underestimates clinic TT. DES model outputs are dependent on input data, and the potential impact on wait times and crowding can be overestimated because models are simplifications of the real-world system [49].

### Implications for Research, Policy, and Practice

Strategies such as configurations on consultation start times and patient arrival can be considered in outpatient clinic settings with distinct patient groups who share the same resources, such as in dual practice systems to improve wait times and reduce crowding. Although healthcare facilities differ in process flows, facility setup, and practices, simulation can aid in determining areas for improvement in process flow or patient scheduling, leading to better patient care experience through shorter wait times or increased service efficiency. Future research can focus on the feasibility of implementation, patient/healthcare provider acceptability, and changes to resource availability to determine if additional resources can offer the same or better reduction in wait times or crowd control. Additionally, qualitative study to obtain narrative inputs from healthcare providers of the clinic would provide valuable insights for patient flow optimisation.

Although the dual practice system in MOH Malaysia is thought to improve specialist retention and enable private patients to gain benefits such as the ability to choose their preferred specialists and appointment time allocations [2], dual practice systems may lead to unintended effects or have negative impacts that may outweigh the positive [3,6,50,51]. Thus, there is a need for continuous monitoring and evaluation by hospital managers and the MOH to ensure benefits are gained while maintaining optimal public service provision.

As for the wait time gap between public and private patients in a dual practice system setting, healthcare policymakers should consider the benefits, risks, and implications of this system towards outcomes on healthcare access (wait times), utilisation, and resource use, and ensure that effective strategies are implemented to improve timely provision of outpatient services for patients regardless of status. The study has demonstrated that DES can be used to identify strategies to reduce wait times and crowding in an outpatient setting with a dual practice system; this can be leveraged to assist healthcare policymakers in formulating evidence-based policies for outpatient service enhancement.

## 5. Conclusions

Although public patients had longer wait times than private patients based on registration and clinic turnaround times in a dual practice outpatient setting, changing consultation start time to match patient arrival time and implementing staggered patient arrival can potentially improve both public and private patients’ wait times and reduce crowding, especially during clinic peak hours without incurring additional need for resources. The greater reduction in wait times for public patients compared to private patients would narrow the wait time gap between public and private patients. Healthcare managers and policymakers should consider using simulation approaches as a tool for continuous monitoring and improvement of healthcare operational efficiency to meet rising healthcare demand and costs.

## Figures and Tables

**Table 1:** Operational characteristics and patient arrival for base case model and scenario analyses.

Simulation	Arrival Pattern	QMS Registration Start Time	Consultation Start Time	Maximum Number of Public Patient Arrival in Every 30-min Slot	Last Public Patient Time Slot	Private Patients’ Start Time	Maximum Number of Private Patient Arrival in Every 30-min Slot
Base case	Random	TMS (7:00 a.m.)	TMS (~9:00 a.m.)	TMS (22)	~12:00 p.m.	TMS (11:30 a.m.)	TMS (9)
Scenario 1	Even	TMS (7:00 a.m.)	TMS (~9:00 a.m.)	TMS (22)	~12:00 p.m.	TMS (11:30 a.m.)	TMS (9)
Scenario 2	Even	TMS (7:00 a.m.)	8:15 a.m.	TMS (22)	~12:00 p.m.	TMS (11:30 a.m.)	TMS (9)
Scenario 3	Even	7:30 a.m.	8:15 a.m.	10	12:00 p.m.	1:30 p.m.	2
Scenario 4	Even	7:30 a.m.	TMS (~9:00 a.m.)	10	12:00 p.m.	1:30 p.m.	2
Scenario 5	Even	8:00 a.m.	TMS (~9:00 a.m.)	7	3:00 p.m.	TMS (11:30 a.m.)	TMS (9)
Scenario 6	Even	8:00 a.m.	TMS (~9:00 a.m.)	10	12:30 p.m.	1:30 p.m.	2
Scenario 7	Even	8:00 a.m.	TMS (~9:00 a.m.)	10	12:30 p.m.	11:00 a.m.	2

QMS: general outpatient registration counter; TMS: time-motion study.

**Table 2:** Process times and turnaround times based on TMS.

Median (Q1–Q3) Time (Hours: Min)			
Observation 1	Public (*n* = 338)		Private (*n* = 32)
	Obs (*n* = 191)	Gyn (*n* = 147)	
Registration TT ^a, b^	00:45 (00:31–00:55)	00:29 (00:22–00:38)	00:14 (00:12–00:16)
Registration and payment ^b^	-	-	00:04 (00:02–00:07)
Observation 2	Public (*n* = 357)		Private (*n* = 32)
	Obs (*n* = 180)	Gyn (*n* = 177)	
Clinic TT ^a^	01:39 (01:15–02:08)	01:33 (01:11–01:54)	01:06 (00:43–01:33)
Vital sign measurement	00:02 (00:01–00:03)	00:02 (00:01–00:04)	00:01 (00:01–00:01)
Laboratory ^c^	00:04 (00:03–00:06)	-	00:03 (00:02–00:04)
Consultation ^d^	00:13 (00:10–00:20)	00:10 (00:07–00:17)	00:17 (00:13–00:21)
Appointment setting	00:02 (00:01–00:04)	00:02 (00:01–00:04)	00:02 (00:01–00:05)

TT: turnaround time. ^a^ Kruskal–Wallis H test, statistically significant, *p* < 0.01. ^b^ Registration processes for private patients were observed during TMS Observation 2 period. ^c^ Gyn: *n* = 2 (excluded from analysis), Obs: *n* = 51 and Private: *n* = 21. ^d^ Mann–Whitney Test (between Obs public and private patients (as the majority of private patients were Obs patients (*n* = 29, 91%))), statistically significant, *p* = 0.021.

## Data Availability

Data that support the findings of this study are available from the Ministry of Health Malaysia, but restrictions apply to the availability of these data and so are not publicly available. The data, however, can be obtained from the corresponding author and Head of Centre for Biostatistics and Data Repository, National Institutes of Health, Ministry of Health Malaysia on reasonable request and with permission from the Director-General of Health Malaysia.

## Supplementary Materials

The following are available online at https://www.mdpi.com/article/10.3390/healthcare10020189/s1, Table S1: Average number of public and private patients per day (October 2017), Figure S1: Patient selection flow chart for TMS Observation 1, Figure S2: Patient selection flow chart for TMS Observation 2, Table S2: DES model input parameters, Table S3: Patient arrival data for TMS, DES base case, and DES scenario simulation, Figure S3: DES model layout of QMS, private patient, and revenue counters at the outpatient department, Figure S4: DES model layout of the O&G specialist outpatient clinic, Table S4: Overall TT based on even and random arrival distributions in base case model simulation, Table S5: Base case and scenario simulation outputs on overall TT, Table S6: Base case and scenario simulation outputs on the number of patients at the O&G clinic waiting area per hour.
