# Supplementary material for: Applying Discrete Event Simulation to Reduce Patient Wait Times and Crowding: The Case of a Specialist Outpatient Clinic with Dual Practice System
Source: Healthcare (Basel). 2022 Jan 19;10(2):189. doi: 10.3390/healthcare10020189 (PMC8871892; doi:10.3390/healthcare10020189)
Supplement: Supplementary file 1 [file healthcare-10-00189-s001.zip › healthcare-1536724-supplementary.pdf]

healthcare
MDPI

Supplementary Materials

Table S1. Average number of public and private patients per day (October 2017).

Date	Day	Public		Private
		Obs	Gyn	
2 October 2017	Monday	19	101	5
3 October 2017	Tuesday	73	12	1
4 October 2017	Wednesday	90	19	7
5 October 2017	Thursday	43	95	10
6 October 2017	Friday	2	10	-
9 October 2017	Monday	14	91	12
10 October 2017	Tuesday	73	16	4
11 October 2017	Wednesday	91	10	8
12 October 2017	Thursday	28	82	9
13 October 2017	Friday	8	15	-
16 October 2017	Monday	24	102	11
17 October 2017	Tuesday	38	7	-
19 October 2017	Thursday	15	22	4
20 October 2017	Friday	4	4	-
23 October 2017	Monday	16	98	10
24 October 2017	Tuesday	100	10	1
25 October 2017	Wednesday	111	13	4
26 October 2017	Thursday	41	90	6
27 October 2017	Friday	7	8	-
30 October 2017	Monday	16	103	7
31 October 2017	Tuesday	81	5	-
Average		43	43	7

Note: 18 October 2017 (Wednesday) was a public holiday in Malaysia.

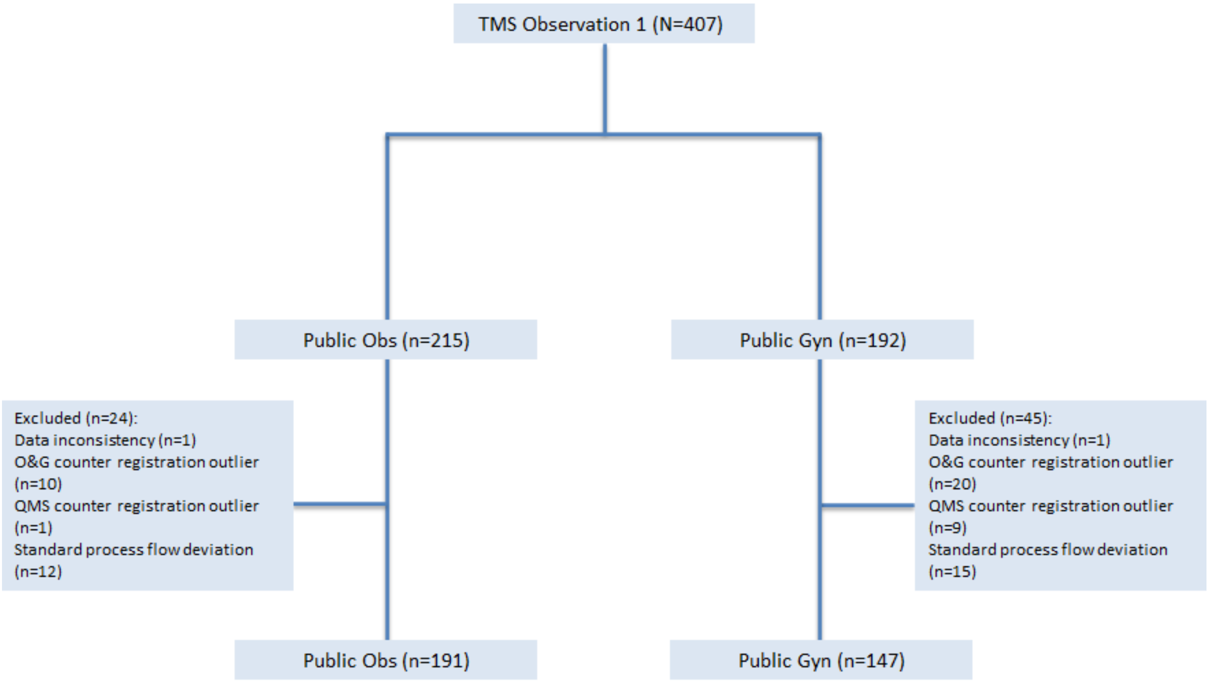

Figure S1. Patient selection flow chart for TMS Observation 1.

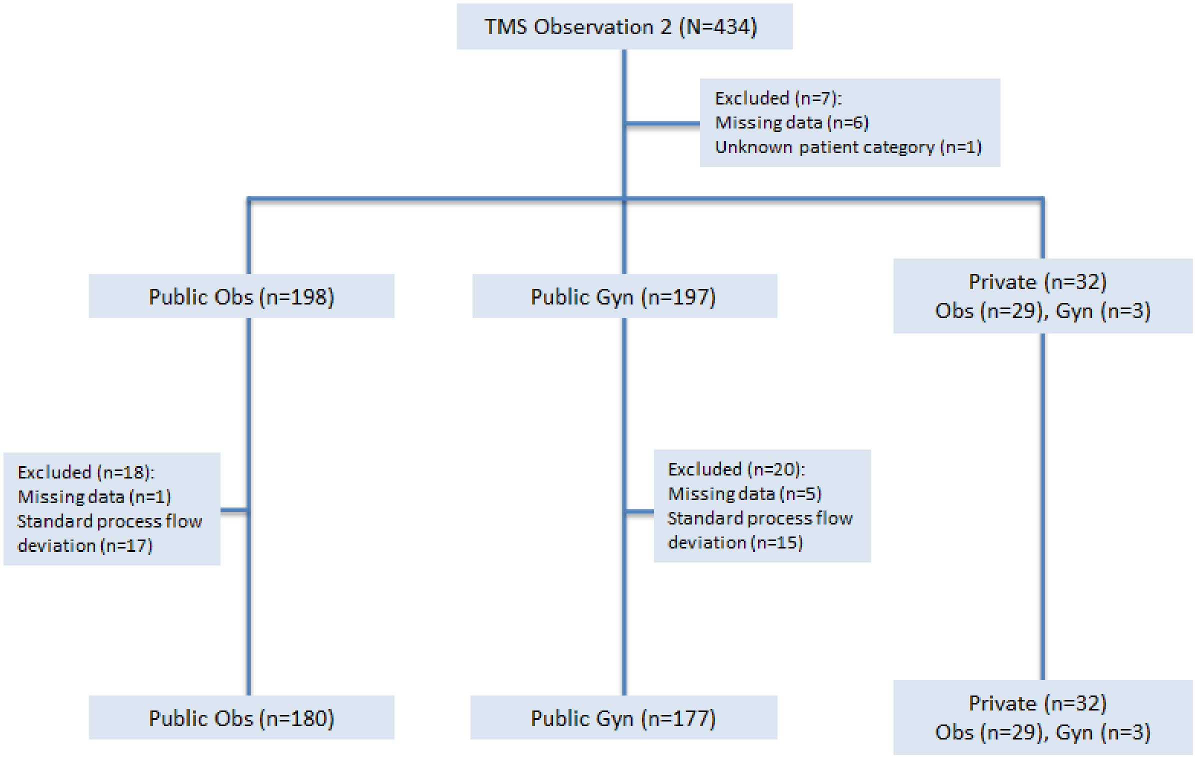

Figure S2. Patient selection flow chart for TMS Observation 2.

healthcare MDPI

Table S2. DES model input parameters.

	Parameter	Probability Distribution (Model Input Value in Minutes)				Source
		Obs	Gyn	Private	General	
1	Registration					
	QMS counter processing time (minimum, maximum, median)	triangular (0.1, 0.5, 0.3)	triangular (0.1, 0.5, 0.3)	n/a	triangular (0.1, 0.5, 0.3)	TMS (Observation 1)
	Private patient counter processing time	n/a	n/a	Johnson-bounded (0.38, 15.85, 0.98, 0.79)	n/a	TMS (Observation 1)
2	Revenue counter					
	Revenue counter processing time	Non-elderly (100%)<br>Elderly (0%)<br>triangular (1, 3, 1.4)	Non-elderly (86.3%)<br>Elderly (13.7%)<br>triangular (1, 3, 1.4)	n/a	Non-elderly (66.1%)<br>Elderly (33.9%)<br>triangular (1, 3, 1.4)	TMS (Observation 1) with expert opinion
3	O&G counter					
	O&G counter	triangular (1, 2,	triangular (1, 2,	uniform (0.5, 1.5)	n/a	Expert opinion

	Parameter	Probability Distribution (Model Input Value in Minutes)							Source
		Obs		Gyn		Private		General	
	processing time		1.4)		1.4)				
4	Vital sign measurement station and laboratory								
	Vital sign measurement station processing time	New (41.1%)	Empirical	New (31%)	Empirical		Empirical	n/a	TMS (Observation 2)
		Follow-up (58.9%)	Empirical	Follow-up (69%)	Empirical				TMS (Observation 2)
	Laboratory processing time	New (27.5%)	loglogistic (0.00, 15.50, 2.67)	New (100%)	loglogistic (0.00, 15.50, 2.67)		Empirical	n/a	TMS (Observation 2)
		Follow-up (72.5%)	loglogistic (0.07, 8.90, 2.64)	Follow-up (0%)	-				TMS (Observation 2)
5	Consultation								
	Case review and management		triangular (6, 10, 8)		triangular (6, 10, 8)			n/a	Expert opinion
	Doctor and specialist discussion	Yes (10%)	triangular (5, 10, 8)	Yes (10%)	triangular (5, 10, 8)	All cases	pearsont6 (9.43, 25.45, 2.04, 6.61)	n/a	Expert opinion
		No (90%)	n/a	No (90%)	n/a				Expert opinion

Parameter	Probability Distribution (Model Input Value in Minutes)						Source
	Obs		Gyn		Private	General	
Consultation time	New (41.1%)	lognormal2 (0.00, 15.50, 0.53)	New (31%)	loglogistic (0.00, 15.50, 2.67)		n/a	TMS (Observation 2)
	Follow-up (58.9%)	loglogistic (3.42, 9.20, 2.21)	Follow-up (69%)	loglogistic (0.07, 8.90, 2.64)			TMS (Observation 2)
Appointment setting							
6 Nurses' station processing time	New (41.1%)	Empirical	New (31%)	Empirical	Empirical	n/a	TMS (Observation 2)
	Follow-up (58.9%)	Empirical	Follow-up (69%)	Empirical			TMS (Observation 2)

Note: Probability distributions used: loglogistic: log logistic; lognormal2: log normal; pearsont6: Pearson type VI; johnsonbounded: Johnson Single Bounded. N/a: not applicable.

Table S3. Patient arrival data for TMS, DES base case, and DES scenario simulation.

	Number of Patient Arrival Per 30-min Slot Based on THIS and TMS Data and in DES Base Case					Total Patients Per 30-min Slot	Number of Patient Arrival Per 30-min Slot in Scenario Simulation							
	**Start time**	**General**	**Obs**	**Gyn**	**Private**		**Obs: 7 Patients**	**Obs: 10 Patients (7:30 a.m.)**	**Obs: 10 Patients (8:00 a.m.)**	**Gyn: 7 Patients**	**Gyn: 10 Patients (7:30 a.m.)**	**Gyn: 10 Patients (8:00 a.m.)**	**Private: 2 Patients (11:00 a.m.)**	**Private: 2 Patients (1:30 p.m.)**
Day 1	7:00 a.m.	59	0	1	0	60	0	0	0	0	0	0	0	0
	7:30 a.m.	74	3	9	0	86	0	2	0	0	10	0	0	0
	8:00 a.m.	88	2	9	0	99	2	2	2	7	10	10	0	0
	8:30 a.m.	86	0	15	0	101	2	2	2	7	10	10	0	0
	9:00 a.m.	80	2	8	0	90	2	2	2	7	10	10	0	0
	9:30 a.m.	77	3	15	0	95	2	2	2	7	10	10	0	0
	10:00 a.m.	71	1	14	0	86	2	2	2	7	10	10	0	0
	10:30 a.m.	62	1	11	0	74	2	2	2	7	10	10	0	0
	11:00 a.m.	30	1	6	0	37	2	0	2	7	10	10	2	0
	11:30 a.m.	9	1	1	0	11	0	0	0	7	9	10	2	0
	12:00 p.m.	11	0	0	0	11	0	0	0	7	0	9	2	0
	12:30 p.m.	2	0	0	0	2	0	0	0	7	0	0	2	0
	1:00 p.m.	4	0	0	0	4	0	0	0	0	0	0	0	0
	1:30 p.m.	64	0	0	0	64	0	0	0	7	0	0	2	2
	2:00 p.m.	67	0	0	8	75	0	0	0	7	0	0	1	2
	2:30 p.m.	45	0	0	3	48	0	0	0	5	0	0	0	2
	3:00 p.m.	25	0	0	0	25	0	0	0	0	0	0	0	2
	3:30 p.m.	5	0	1	0	6	0	0	0	1	1	1	0	2
	4:00 p.m.	7	0	0	0	7	0	0	0	0	0	0	0	1
Day 2	7:00 a.m.	70	2	1	0	73	0	0	0	0	0	0	0	0
	7:30 a.m.	93	5	0	0	98	0	10	0	0	2	0	0	0
	8:00 a.m.	134	5	2	0	141	7	10	10	2	2	2	0	0
	8:30 a.m.	133	15	4	0	152	7	10	10	2	2	2	0	0

	Number of Patient Arrival Per 30-min Slot Based on THIS and TMS Data and in DES Base Case					Total Patients Per 30-min Slot	Number of Patient Arrival Per 30-min Slot in Scenario Simulation							
	Start time	General	Obs	Gyn	Private		Obs: 7 Patients	Obs: 10 Patients (7:30 a.m.)	Obs: 10 Patients (8:00 a.m.)	Gyn: 7 Patients	Gyn: 10 Patients (7:30 a.m.)	Gyn: 10 Patients (8:00 a.m.)	Private: 2 Patients (11:00 a.m.)	Private: 2 Patients (1:30 p.m.)
	9:00 a.m.	126	14	1	0	141	7	10	10	2	2	2	0	0
	9:30 a.m.	93	14	1	0	108	7	10	10	2	2	2	0	0
	10:00 a.m.	71	2	1	0	74	7	10	10	2	2	2	0	0
	10:30 a.m.	43	5	1	0	49	7	7	10	2	0	2	0	0
	11:00 a.m.	25	4	0	0	29	7	0	7	0	0	0	2	0
	11:30 a.m.	19	1	0	0	20	7	0	0	0	0	0	2	0
	12:00 p.m.	12	0	1	3	16	7	0	0	0	0	0	0	0
	12:30 p.m.	4	0	0	0	4	4	0	0	0	0	0	0	0
	1:00 p.m.	0	0	0	0	0	0	0	0	0	0	0	0	0
	1:30 p.m.	68	0	0	0	68	0	0	0	0	0	0	0	2
	2:00 p.m.	67	0	1	0	68	0	0	0	1	1	1	0	2
	2:30 p.m.	36	1	1	1	39	1	1	1	1	1	1	0	0
	3:00 p.m.	20	0	1	0	21	0	0	0	1	1	1	0	0
	3:30 p.m.	13	0	0	0	13	0	0	0	0	0	0	0	0
	4:00 p.m.	3	1	0	0	4	1	1	1	0	0	0	0	0
Day 3	7:00 a.m.	64	4	0	0	68	0	0	0	0	0	0	0	0
	7:30 a.m.	61	4	2	0	67	0	10	0	0	2	0	0	0
	8:00 a.m.	94	8	0	0	102	7	10	10	2	2	2	0	0
	8:30 a.m.	107	11	1	0	119	7	10	10	2	2	2	0	0
	9:00 a.m.	86	19	4	0	109	7	10	10	2	2	2	0	0
	9:30 a.m.	69	16	2	0	87	7	10	10	2	2	2	0	0
	10:00 a.m.	57	12	0	0	69	7	10	10	2	0	2	0	0
	10:30 a.m.	34	7	1	0	42	7	10	10	0	0	0	0	0
	11:00 a.m.	25	5	0	0	30	7	10	10	0	0	0	2	0
	11:30 a.m.	11	3	0	0	14	7	10	10	0	0	0	2	0

	Number of Patient Arrival Per 30-min Slot Based on THIS and TMS Data and in DES Base Case					Total Patients Per 30-min Slot	Number of Patient Arrival Per 30-min Slot in Scenario Simulation							
	**Start time**	**General**	**Obs**	**Gyn**	**Private**		**Obs: 7 Patients**	**Obs: 10 Patients (7:30 a.m.)**	**Obs: 10 Patients (8:00 a.m.)**	**Gyn: 7 Patients**	**Gyn: 10 Patients (7:30 a.m.)**	**Gyn: 10 Patients (8:00 a.m.)**	**Private: 2 Patients (11:00 a.m.)**	**Private: 2 Patients (1:30 p.m.)**
	12:00 p.m.	5	1	0	2	8	7	0	10	0	0	0	2	0
	12:30 p.m.	10	0	0	0	10	7	0	0	0	0	0	2	0
	1:00 p.m.	4	0	0	0	4	0	0	0	0	0	0	0	0
	1:30 p.m.	18	0	0	0	18	7	0	0	0	0	0	0	2
	2:00 p.m.	33	0	0	6	39	7	0	0	0	0	0	0	2
	2:30 p.m.	22	0	0	0	22	6	0	0	0	0	0	0	2
	3:00 p.m.	10	0	0	0	10	0	0	0	0	0	0	0	2
	3:30 p.m.	12	0	0	0	12	0	0	0	0	0	0	0	0
	4:00 p.m.	5	0	0	0	5	0	0	0	0	0	0	0	0
Day 4	7:00 a.m.	54	0	4	0	58	0	0	0	0	0	0	0	0
	7:30 a.m.	105	1	5	0	111	0	3	0	0	10	0	0	0
	8:00 a.m.	120	2	9	0	131	3	3	3	7	10	10	0	0
	8:30 a.m.	124	0	13	0	137	3	3	3	7	10	10	0	0
	9:00 a.m.	127	5	9	0	141	3	3	3	7	10	10	0	0
	9:30 a.m.	128	6	9	0	143	3	3	3	7	10	10	0	0
	10:00 a.m.	115	3	14	0	132	3	3	3	7	10	10	0	0
	10:30 a.m.	96	4	13	0	113	3	3	3	7	10	10	0	0
	11:00 a.m.	54	1	3	0	58	3	2	3	7	10	10	2	0
	11:30 a.m.	23	1	2	0	26	2	0	2	7	1	10	2	0
	12:00 p.m.	14	0	0	4	18	0	0	0	7	0	1	2	0
	12:30 p.m.	10	0	0	0	10	0	0	0	7	0	0	2	0
	1:00 p.m.	2	0	0	0	2	0	0	0	0	0	0	0	0
	1:30 p.m.	37	1	0	1	39	1	1	1	7	0	0	1	2
	2:00 p.m.	35	1	0	3	39	1	1	1	4	0	0	0	2
	2:30 p.m.	28	0	0	1	29	0	0	0	0	0	0	0	2

	Number of Patient Arrival Per 30-min Slot Based on THIS and TMS Data and in DES Base Case					Total Patients Per 30-min Slot	Number of Patient Arrival Per 30-min Slot in Scenario Simulation							
	**Start time**	**General**	**Obs**	**Gyn**	**Private**		**Obs: 7 Patients**	**Obs: 10 Patients (7:30 a.m.)**	**Obs: 10 Patients (8:00 a.m.)**	**Gyn: 7 Patients**	**Gyn: 10 Patients (7:30 a.m.)**	**Gyn: 10 Patients (8:00 a.m.)**	**Private: 2 Patients (11:00 a.m.)**	**Private: 2 Patients (1:30 p.m.)**
	3:00 p.m.	15	0	0	0	15	0	0	0	0	0	0	0	2
	3:30 p.m.	8	0	0	0	8	0	0	0	0	0	0	0	1
	4:00 p.m.	7	0	0	0	7	0	0	0	0	0	0	0	0
Day 5	7:00 a.m.	45	0	0	0	45	0	0	0	0	0	0	0	0
	7:30 a.m.	63	0	0	0	63	0	2	0	0	2	0	0	0
	8:00 a.m.	82	0	3	0	85	2	2	2	2	2	2	0	0
	8:30 a.m.	90	0	2	0	92	2	1	2	2	2	2	0	0
	9:00 a.m.	98	0	2	0	100	1	0	1	2	2	2	0	0
	9:30 a.m.	100	1	2	0	103	0	0	0	2	2	2	0	0
	10:00 a.m.	32	2	3	0	37	0	0	0	2	2	2	0	0
	10:30 a.m.	28	0	0	0	28	0	0	0	2	1	2	0	0
	11:00 a.m.	24	2	0	0	26	0	0	0	1	0	1	0	0
	11:30 a.m.	14	0	1	0	15	0	0	0	0	0	0	0	0
	12:00 p.m.	2	0	0	0	2	0	0	0	0	0	0	0	0
	12:30 p.m.	0	0	0	0	0	0	0	0	0	0	0	0	0
	1:00 p.m.	1	0	0	0	1	0	0	0	0	0	0	0	0
	1:30 p.m.	0	0	0	0	0	0	0	0	0	0	0	0	0
	2:00 p.m.	0	0	0	0	0	0	0	0	0	0	0	0	0
	2:30 p.m.	19	0	0	0	19	0	0	0	0	0	0	0	0
	3:00 p.m.	23	0	0	0	23	0	0	0	0	0	0	0	0
	3:30 p.m.	7	0	0	0	7	0	0	0	0	0	0	0	0
	4:00 p.m.	5	0	0	0	5	0	0	0	0	0	0	0	0
Total		4358	203	209	32	4802	203	203	203	209	209	209	32	32

Note: A total of 5034 patient arrivals at the outpatient department and O&G clinic were captured in THIS during TMS Observation 2 period. For general patients, out of 4571 patient arrivals captured in THIS, 213 patients were excluded due to duplicate registration and registration time anomalies (arrival after 4:30 p.m. (outpatient clinics close at 5:00 p.m.), arrival out of clinic working hours or day), leaving 4358 general patients for

inclusion in simulation. Out of 463 O&G clinic patient arrivals captured in THIS, 19 patients were excluded due to unknown patient type, missing data, duplicate registration, registration time anomalies, and misclassification (male patient, Obs patient above the age of 50 years). The remaining 444 patients (Obs, n = 203, Gyn, n = 209 and Private, n = 32) were included in the simulation.

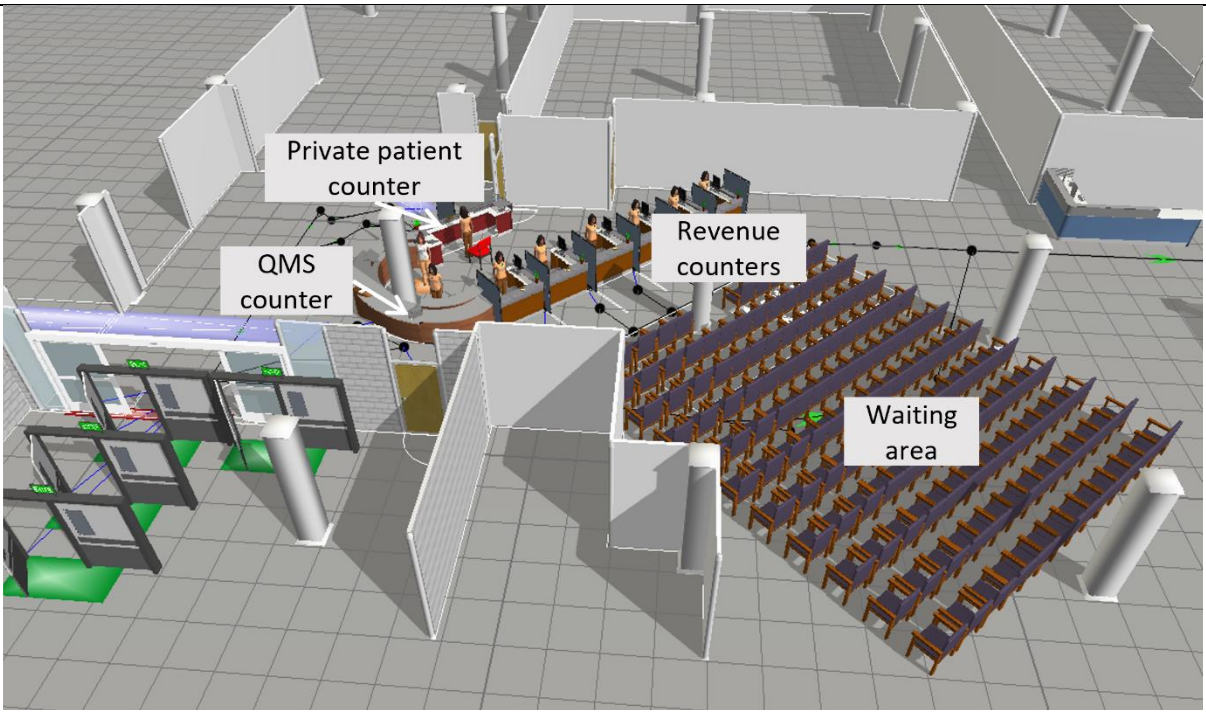

Figure S3. DES model layout of QMS, private patient, and revenue counters at the outpatient department.

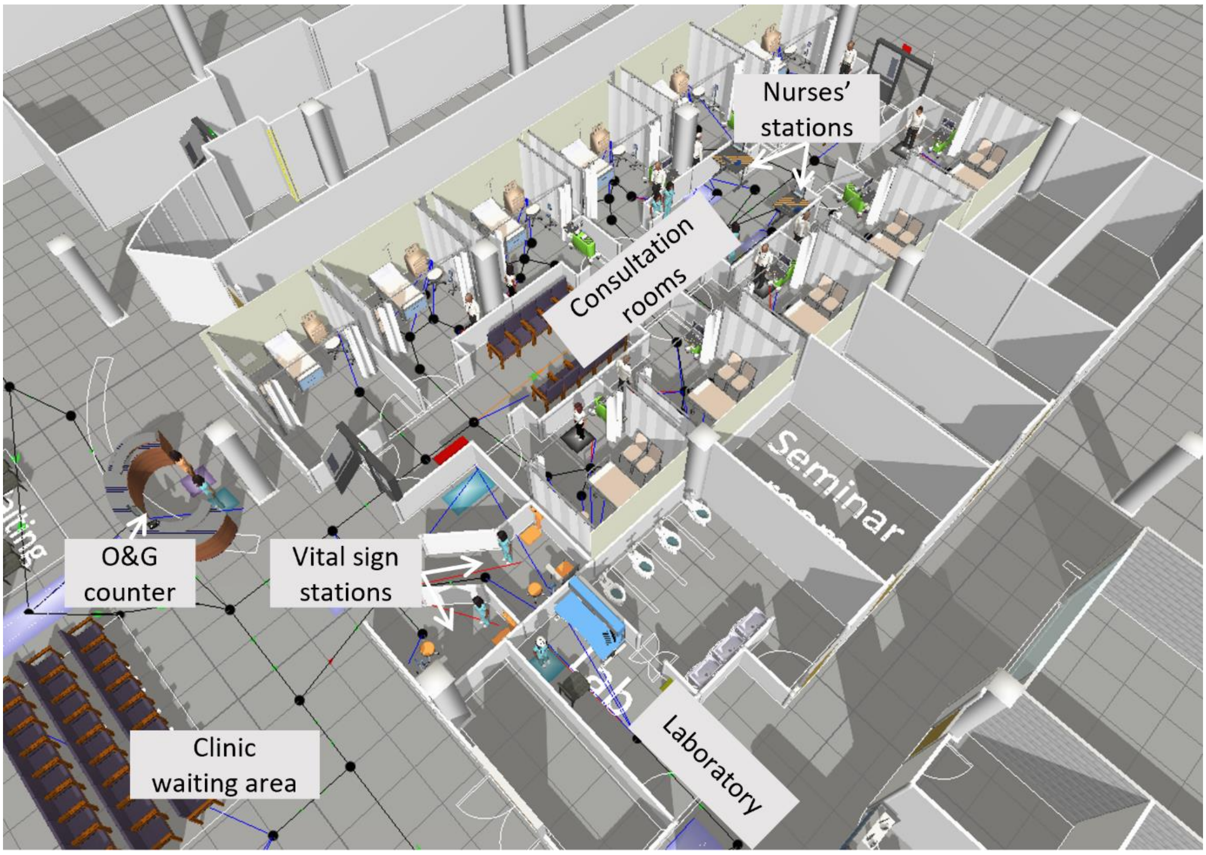

Figure S4. DES model layout of the O&G specialist outpatient clinic.

Table S4. Overall TT based on even and random arrival distributions in base case model simulation.

Patient Type	Overall TT, Median (Q1-Q3) (Hours: Min)	
	Base Case (Even Distribution)	Base Case (Random Distribution)
Public Obs	02:11 (01:47–02:45)	02:08 (01:42–02:37)
Public Gyn	01:45 (01:20–02:10)	01:42 (01:17–02:07)
Private	01:38 (01:09–02:21)	01:37 (01:07–02:15)

TT: turnaround time.

Table S5. Base case and scenario simulation outputs on overall TT.

Simulation	Overall TT, Median (Q1-Q3) (Hours: Min)		
	Public		Private
	Obs	Gyn	
Base case	02:08 (01:42–02:37)	01:42 (01:17–02:07)	01:37 (01:07–02:15)
Scenario 1	01:48 (01:27–02:10)	01:36 (01:07–01:50)	01:21 (00:59–02:00)
Scenario 2	01:22 (01:06–01:46)	01:05 (00:49–01:22)	01:22 (01:00–02:02)
Scenario 3	01:18 (01:05–01:36)	01:01 (00:50–01:13)	01:16 (00:57–01:39)
Scenario 4	01:49 (01:34–02:08)	01:32 (01:10–01:45)	01:16 (00:57–01:41)
Scenario 5	01:10 (00:57–01:34)	00:56 (00:44–01:17)	01:44 (01:08–02:22)
Scenario 6	01:24 (01:08–01:45)	01:06 (00:52–01:22)	01:32 (01:06–01:58)
Scenario 7	01:25 (01:10–01:53)	01:07 (00:54–01:22)	02:03 (01:26–02:51)

TT: turnaround time.

Table S6. Base case and scenario simulation outputs on the number of patients at the O&G clinic waiting area per hour.

Time	Average Number of Patients at the O&G Clinic Waiting Area Per Hour							
	Base Case	Scenario 1	Scenario 2	Scenario 3	Scenario 4	Scenario 5	Scenario 6	Scenario 7
7:00 a.m.–8:00 a.m.	0	0	0	0	0	0	0	0
8:00 a.m.–9:00 a.m.	19	20	20	24	24	10	13	13
9:00 a.m.–10:00 a.m.	36	37	25	26	39	21	28	28
10:00 a.m.–11:00 a.m.	41	40	30	24	36	18	26	26
11:00 a.m.–12:00 p.m.	46	39	30	27	36	22	30	33

12:00 p.m.–1:00 p.m.	40	27	22	19	25	27	28	35
1:00 p.m.–2:00 p.m.	17	6	5	3	5	9	7	13
2:00 p.m.–3:00 p.m.	12	10	10	9	9	27	13	13
3:00 p.m.–4:00 p.m.	6	5	5	6	6	13	8	4
4:00 p.m.–5:00 p.m.	1	1	1	2	2	2	3	1
